# Serologic Evidence of West Nile Virus Transmission, Jamaica, West Indies

**DOI:** 10.3201/eid0907.030249

**Published:** 2003-07

**Authors:** Alan P. Dupuis, Peter P. Marra, Laura D. Kramer

**Affiliations:** * New York State Department of Health, Slingerlands, New York, USA; † Smithsonian Environmental Research Center, Edgewater, Maryland, USA

**Keywords:** arbovirus, birds, Caribbean, Jamaica, Mexico, neutralizing antibodies, Puerto Rico, St. Louis encephalitis, West Nile virus, dispatch

## Abstract

In spring 2002, an intensive avian serosurvey was initiated in Jamaica, Puerto Rico, and Mexico. We collected >1,600 specimens from resident and nonresident neotropical migratory birds before their northerly migrations. Plaque reduction neutralization test results indicated specific neutralizing antibodies to West Nile virus in 11 resident species from Jamaica.

West Nile virus (WNV) is maintained in nature between birds and *Culex* species mosquitoes (*1*,*2*). Unlike other viruses maintained in bird and mosquito transmission cycles (for example, St. Louis encephalitis, western equine encephalomyelitis, and eastern equine encephalomyelitis), the WNV strain responsible for the current epizootic in the Western Hemisphere is associated with a high number of avian deaths (*3*). Although crows and other corvids appear to be especially susceptible to disease (*4*), WNV has been documented in >190 bird species, including neotropical migratory species and exotic zoo specimens (*5*).

Birds have been implicated in spreading WNV during migratory events in Europe, Asia, Africa, and the Middle East (*6*–*9*). Because of the apparent ease of infecting a multitude of avian hosts, WNV can potentially be introduced during annual migratory events in the Western Hemisphere. Predicting the incursion of WNV into the tropics is complicated by our incomplete knowledge regarding geographic connectivity in populations of migratory birds between winter and summer. We do not know where most species of migratory birds from North America spend their winter, where wintering birds spend their summer, or the routes they use while in transit. Evidence from mark-and-recapture efforts suggests that birds from the northeastern United States tend to winter in the southeastern United States and Greater Antilles (e.g., Puerto Rico, Jamaica) whereas birds from the western United States migrate to Mexico and Central America (*10*–*12*). In response to the potential introduction of WNV in tropical America during the fall migrations, we established a network of monitoring sites on the overwintering grounds of neotropical migratory birds in Jamaica, Puerto Rico, and Mexico.

## The Study

The primary goal of the monitoring system was to obtain a large number of blood specimens from birds belonging to as many species as possible across the Caribbean. At seven study sites in Jamaica, Puerto Rico, and the Yucatan Peninsula of Mexico, we erected 15 mist nets (12-m) daily for 3 to 4 weeks during January through March 2002. We collected 1,619 avian blood specimens, which represented 98 species, 25 families, and eight orders. In Jamaica, 542 samples were collected from Westmoreland, Manchester, and St. Catherine Parishes; 649 samples were collected at Roosevelt Roads Naval Station in Puerto Rico; and 430 samples were collected from the states of Yucatan and Campeche in Mexico (Table 1). At the time of capture, all migratory birds were banded with an aluminum U.S. Fish and Wildlife Service band and 3–5 breast feathers were removed for isotope analysis. Resident birds had outer rectrices cut. All birds were evaluated for age and gender if possible, bled with microcapillary tubes from the brachial vein, and released. Blood was added immediately to BA-1 medium, consisting of M199 medium with Hank’s salts, 1% bovine albumin, TRIS base (tris [hydroxymethyl] aminomethane), sodium bicarbonate, 20% fetal bovine serum (FBS), and antibiotics. The samples were placed in a cooler on ice packs until storage in a –20°C freezer. Samples were sent on dry ice or hand-carried on ice packs to the Arbovirus Laboratories, Wadsworth Center, New York State Department of Health for serologic analysis and virus isolation attempts.

**Table 1:** Number of birds collected at seven field sites in the Caribbean

Field site	Migratory birds	Resident birds	Total	Flavivirus positives (%)^a^	
Westmoreland Parish, Jamaica	156	232	388	18 (4.6)	
Manchester Parish (site 1), Jamaica	2	21	23	1 (4.4)	
Manchester Parish (site 2), Jamaica	24	83	107	4 (3.7)	
St. Catherine Parish, Jamaica	12	12	24	4 (16.7)	
Yucatan, Mexico	70	102	172	2 (1.2)	
Campeche, Mexico	114	144	258	0 (0.0)	
Mexico totals	184	246	430	2 (0.5)	
Puerto Rico (one collection site) totals	391	256	647	5 (1.1)^b^	
Jamaica totals	194	348	542	27 (5.0)	
Totals	769	850	1,619	34 (2.4)^c^	

^a^Based on screening enzyme-linked immunosorbent assay (ELISA) results. ^b^Results based on 441 samples suitable for ELISA (206 samples were blood clots only). ^c^Results based on 1,413 samples tested by ELISA.

Specimens were screened at 1:100 for antibodies against flaviviruses by using an indirect enzyme-linked immunosorbent assay (ELISA) (*13*). Samples with a P/N ratio >2.0 were tested further by a plaque reduction neutralization test (PRNT) for Ilhéus virus (ILHV), St. Louis encephalitis virus (SLEV), and WNV, as described (*14*). The particular virus strains used for the PRNTs were ILHV (original), SLEV 59268 Parton, and WNV (3100365), an isolate from a pool of *Culex* sp. mosquitoes collected in Staten Island, New York. The indirect ELISA was chosen to screen the samples in order to take advantage of its ability to detect antibodies against a wide range of flaviviruses. PRNT was used as a confirmatory assay to differentiate among recognized flaviviruses (*15*,*16*). Briefly, serial dilutions of test samples were mixed with an equal amount of virus suspension containing 200 PFU/1 mL and incubated at 37°C for 1 h. We then added 0.05 mL of each virus-diluted blood sample onto 1 well of a 12-well tissue culture plate containing confluent monolayers of African green monkey kidney cells (Vero). The plate was incubated for 1 h at 37°C, after which an agarose overlay was added and incubation was continued. When virus plaques became visible, we added a second overlay containing neutral red and counted plaques. The antibody titer reported is the dilution of serum that inhibited 90% of the test virus inoculum. For virus isolation attempts using confluent Vero cell monolayers, 0.1 mL of each serum specimen was added onto one well of a six-well tissue culture plate, incubated for 1 h, rinsed with phosphate-buffered saline, and then the cells were refed with minimum essential medium containing 2% FBS. Cells were monitored once a day for 5 days for cytopathic effect. Cell cultures showing any abnormal cell morphology were then blind passaged after 5 days.

ELISA results indicated 34 of 1,413 serum specimens tested from the three study sites contained immunoglobulin G antibody against a flavivirus. Of the 34 reactive samples representing 20 bird species, 27 were collected in Jamaica (26 residents, 1 migrant), 5 in Puerto Rico (3 residents, 2 migrants), and 2 in Mexico (1 resident, 1 migrant). PRNTs on the Jamaican bird samples indicated 18 WNV infections, 3 SLEV infections, 5 undetermined flavivirus infections (positive results for two or more viruses without a fourfold difference in antibody titer); one additional reactive serum sample was negative for the three viruses tested. Results on the serum samples collected in Puerto Rico indicated one WNV infection in a migratory bird and one SLEV infection in a resident bird; three additional reactive serum samples were negative for all three viruses tested. In Mexico, we found evidence of WNV infection in one migrant bird and SLEV infection in one resident bird (Table 2). Virus isolation attempts were negative for all 1,603 specimens tested (16 samples destroyed). Negative isolation results were not entirely unexpected, considering that birds are viremic for a short period of time (*17*) and maintaining a proper cold chain (i.e., temperatures) to preserve virus is difficult when working in the tropics.

**Table 2:** Ninety percent plaque reduction neutralization titers to West Nile virus, St. Louis encephalitis virus, and Ilhéus virus in enzyme-linked immunosorbent assay reactive bird specimens^a^

Field site	Species	ILHV	SLEV	WNV	Interpretation	
Westmoreland Parish, Jamaica	White-chinned thrush (*Turdus aurantius*)^b^	40	80	640	WNV	
	White-chinned thrush *(T. aurantius*)^b^	<40	<40	320	WNV	
	Jamaican elaenia (*Myiopagis cotta*)^b^	<40	160	640	WNV	
	Bananaquit (*Coereba flaveola*)^b^	<40	80	160	Flavivirus	
	Loggerhead kingbird (*Tyrannus caudifasciatus*)^b^	<40	<40	320	WNV	
	Bananaquit (*C. flaveola*)^b^	<40	<40	160	WNV	
	White-chinned thrush (*Turdus aurantius*)^b^	<40	40	>1,280	WNV	
	Bananaquit *(C. flaveola*)^b^	<40	<40	80	WNV	
	Northern mockingbird (*Mimus polyglottos*)^b^	<40	<40	320	WNV	
	Caribbean dove (*Leptotila jamaicensis*)^b^	<40	>1,280	80	SLEV	
	Jamaican elaenia (*Myiopagis cotta*)^b^	<40	<40	160	WNV	
	Bananaquit (*C. flaveola*)^b^	<40	40	80	Flavivirus	
	White-chinned thrush (*Turdus aurantius*)^b^	<40	40	640	WNV	
	Jamaican elaenia (*Myiopagis cotta*)^b^	<40	80	<40	SLEV	
	Common ground-dove (*Columbina passerina*)^b^	<40	<40	160	WNV	
	Caribbean dove (*Leptotila jamaicensis*)^b^	<40	<40	<40	Negative	
	Black-faced grassquit (*Tiaris bicolor*)^b^	<40	<40	320	WNV	
	Jamaican vireo (*Vireo modestus*)^b^	<40	<40	80	WNV	
Manchester Parish (site 1), Jamaica	Caribbean dove (*Leptotila jamaicensis*)^b^	<40	<40	80	WNV	
Manchester Parish (site 2), Jamaica	Black-faced grassquit (*Tiaris bicolor*)^b^	<40	<40	160	WNV	
	Jamaican oriole (*Icterus leucopteryx*)^b^	<40	160	>1,280	WNV	
	White-eyed thrush (*Turdus jamaicensis*)^b^	<40	<40	320	WNV	
	Orangequit (*Euneornis campestris*)^b^	<40	<40	40	Flavivirus	
St. Catherine Parish, Jamaica	Greater Antillean grackle (*Quiscalus niger*)^b^	40	160	160	Flavivirus	
	Loggerhead kingbird (*Tyrannus caudifasciatus*)^b^	80	320	320	Flavivirus	
	Golden warbler (*Dendroica petechia* sp. )^c^	<40	80	<40	SLEV	
	Northern waterthrush (*Seiurus noveboracensis*)^d^	<40	40	320	WNV	
Yucatan State, Mexico	Caribbean dove (*Leptotila jamaicensis*)^b^	<40	>=1280	80	SLEV	
	Yellow warbler (*Dendroica petechia*)^d^	<40	40	320	WNV	
Roosevelt Roads Naval Station, Puerto Rico	Prairie warbler (*Dendroica discolor*)^d^	<40	<40	<40	Negative	
	Golden warbler (*Dendroica petechia* sp. )^c^	<40	<40	<40	Negative	
	Pearly-eyed thrasher (*Margarops fuscatus*)^b^	<40	<40	<40	Negative	
	Caribbean elaenia (*Elaenia martinica*)^b^	<40	80	<40	SLEV	
	Black and white warbler (*Mniotilta varia*)^c^	<40	40	>1,280	WNV	

^a^ILHV, Ilhéus virus; SLEV, St. Louis encephalitis virus; WNV, West Nile virus. ^b^Resident bird. ^c^Resident yellow warbler subspecies. ^d^Migrant bird.

## Conclusions

We detected neutralizing antibodies to WNV in resident birds from two parishes in Jamaica. This detection marks the earliest evidence of WNV introduction into the neotropics; WNV antibodies have been demonstrated in birds and horses in Mexico (late 2002, spring 2003) (*18*,*19*) and detected in resident birds from the Dominican Republic (spring 2003) (*20*). This evidence of WNV in the neotropics may be an important development in the spread of the virus. The tropics provide all the necessary components (i.e., high temperatures, dense avian populations, and large numbers of *Culex* sp. mosquitoes) to maintain an enzootic focus. Furthermore, the climates of Mexico and the Caribbean are suitable for year-round transmission of the virus. No dead birds have been reported in Jamaica, but surveillance activity there is less intensive than in the United States; the study sites, being rural in nature, are not conducive to observing dead birds. Another contributing factor to the lack of reports of dead birds may be the rapid decomposition of dead birds as a result of the heat, humidity, and detritivore foragers, such as ants.

Arbovirus activity, particularly of flaviviruses, is well documented in the Caribbean and Mexico. Dengue and yellow fever viruses are recurring public health threats in these areas. SLEV is endemic to Mexico and has been isolated from mosquitoes and one Northern mockingbird (*Mimus polyglottos*) nestling in Jamaica (*21*). This disease is still active in the region, and its known range may have expanded into Puerto Rico, considering the one seropositive Caribbean elaenia (*Elaenia martinica*) sampled during this study, although the antibody may have resulted from infection with yet another flavivirus. Neutralizing antibodies to WNV in migratory birds collected in Mexico and Puerto Rico, coupled with the apparent absence of antibody to WNV in the resident bird population, indicate that infection likely occurred in an enzootic area of the United States, but this observation shows that individual birds from at least three species of neotropical migratory birds have survived WNV infection and may serve as hosts for spreading the virus. The results from this study suggest that WNV now appears to be established in Jamaica, on the basis of the neutralizing antibodies to WNV found in the resident bird population.
